# Study of the Prediction of Vibrations in Soft Soil Foundations Based on Field Tests

**DOI:** 10.3390/s24082564

**Published:** 2024-04-17

**Authors:** Jiaxin Lin, Nan Zhang, Yunshi Zhang

**Affiliations:** 1 School of Civil Engineering, Beijing Jiaotong University, Beijing 100044, China; 2 China Electronics Engineering Design Institute Co., Ltd., Beijing 100142, China; 3 Beijing Engineering Research Center for Microvibration Environmental Control, Beijing 100142, China

**Keywords:** microvibration, soft soil foundations, vibration attenuation characteristics, vibration prediction, vibration transmissibility

## Abstract

To explore the prediction of vibrations in soft soil foundations, in light of the construction of laboratories with microvibration requirements on soft soil foundations which are subject to the limitations of urban land planning, field testing was conducted, and the soil surface vibration responses were recorded at different distances from a road under various highway traffic loads. By analyzing the data which summarize the characteristics of soft soil foundations, it is clarified that the vibration response of soft soil foundations mainly occurs at low frequencies, and the vibration response under road traffic loads is prone to resonance at the natural frequency of soft soil foundations. Subsequently, a new vibration prediction method based on the vibration transmission ratio is proposed, and its effectiveness and accuracy based on transmissibility are verified. This research study provides a reference for laboratories constructed on soft soil and for surrounding traffic planning.

## 1. Introduction

In recent years, the high-tech industry has undergone rapid development, with precision machining technology being one of the important directions in high-tech advancement. The adoption of precision machining technology serves as a crucial criterion for assessing a country’s level of high-tech development [1,2]. However, the construction of precision machining laboratories imposes extremely stringent site requirements. Even slight vibrations can significantly impact production and experiments. Therefore, ensuring precision and quality in instrument operation necessitates careful consideration of site selection and vibration control. Microvibration control involves detecting and controlling various environmental vibrations which are widely distributed and transmitted through complex pathways. In practical production and daily life, it is challenging to completely avoid these vibrations, making them one of the limiting factors for many scientific studies and production levels [3,4].

In the first half of the 19th century, simulations of wave propagation in elastic solids began. The initial focus was on the propagation of seismic waves in soil in the field of seismology. Subsequently, this problem gradually extended to other disciplines, leading to more scholars studying the principles of wave propagation. Building upon the foundational research on vibration propagation theory by Lamb [1], an increasing number of scholars have conducted corresponding studies on vibration propagation theory.

Scholars have employed statistical methods to develop predictive models of environmental vibrations [5,6], including research on excitations from rail transit [7,8,9], the exploration of industrial vibration sources [10], and investigations on unique structural environments such as tunnels [11] and laboratories [12,13]. Furthermore, scholars have extensively employed boundary conditions and initial conditions to establish dynamic equilibrium equations of elastic soil foundations for determining the dynamic response of the soil mass. Methods such as the transfer matrix method [14,15], the thin-layer approach [16,17], and soil calculation models employing Green’s functions [18,19] provide valuable insights for analyzing the vibration attenuation characteristics of soils. Currently, vibration propagation has been widely studied using finite element analysis for analytical analysis, often based on experimental data [20,21].

With the advancement of technology, both domestic and international scholars are increasingly focused on the study of microvibrations to prevent adverse effects on sensitive instrument usage in places such as laboratories, hospitals, and electronics factories. In the field of microvibration control, scholars both domestically and internationally are continuously exploring this subject. The main sources of vibration impact can be categorized into external environmental factors and internal vibrations caused by the instrument’s own usage. Highway traffic loads are among the main sources of vibration [22,23], and the vibration source characteristics, site vibration response, and attenuation characteristics are important factors in site selection and microvibration control design. Therefore, accurate evaluation of traffic loads plays a crucial role in the operation and production of large scientific facilities [24,25]. At the same time, soft soil foundations are loose and have low bearing capacities, which can easily lead to significant dynamic deformation and vibration responses in urban transportation, especially in soft soil areas [26,27,28]. To address practical engineering problems and promote in-depth research on microvibration, studying the microvibration of soft soil foundations using highway traffic loads as vibration sources has become a focus of much structural dynamics research.

Soil vibration is closely related to factors such as the type of vehicle, driving speed, mass, road surface characteristics, and traffic flow [29]. Ren et al. [30,31] studied the attenuation patterns of vibrations in different soil types. Liang et al. [32] conducted field measurements and numerical analysis using the ground pulsation measurement method in a certain section of a road-adjacent area. The results showed that the vibration frequencies generated by highway vehicles were in the range from 10 to 20 Hz, and both the vibration intensity and frequency gradually decreased with increasing distance. Hassan [33] conducted in-depth research on the attenuation characteristics of vibrations caused by rail transportation, summarizing the frequency and energy variation characteristics of rail transportation vibration sources with respect to distance and depth. In addition, some scholars have explored the prevention and control of microvibrations. Shi and Ren [34] provided a basis for the anti-microvibration design and subsequent construction of a high-tech factory; they proposed controlling the speed of vehicles and their distance from the factory to avoid adverse effects.

However, these studies still lack specificity, and the unique characteristics of soft soil foundations require researchers to develop a comprehensive theoretical framework. Microvibration control exhibits significant uncertainty, and currently, scholars often analyze environmental vibrations through on-site measurements. By analyzing on-site environmental vibrations, researchers can assess the rationality and reliability of laboratory site selection. Given that site selection often involves avoiding the influence of rail traffic on precision instruments in the laboratory, scholars tend to focus more on the impact of road traffic. Based on the characteristics of soft soil foundations, Wang et al. [35] proposed that it is necessary to clearly identify that soft soil foundations are not universally suitable for constructing high-precision laboratories, and control design should be carried out under the premise of meeting the requirements for minor foundation deformation.

Up to now, there are still many research issues waiting to be explored in the field of microvibration control. Continuous efforts have been made to propose and optimize vibration prediction models for foundations, with Ren et al. [30] proposing a prediction model for the vibration of soft soil foundations based on high-speed train operations. However, this model still has certain limitations. Further in-depth research and exploration are needed for the control of microvibrations in soft soil foundations. To ensure the smooth construction of a precision processing laboratory on a soft soil foundation, this study presents a characteristic analysis of the vibration response induced by different highway traffic loads and proposes a vibration prediction method based on the vibration transmissibility, providing a reference for site selection and laboratory design.

## 2. Project Background

The studied laboratory will be located in a coastal city in China, and the original site is a coastal tidal flat. The proposed construction project had an importance level of Grade I. The geological environment of the construction site had undergone general damage and was considered a moderately complex site, classified as Grade II (moderately complex site). The site contained artificial fill soil, residual soil, and other special types of soil, resulting in a variety of soil types. Therefore, the foundation was classified as Grade II (moderately complex foundation). After reclamation and land transformation, the site is now a flat and spacious construction land. According to on-site investigations and data searches, the current terrain of the site is relatively flat, and there are no obvious adverse geological effects, significant geological disasters, or unconventional buried objects within and surrounding the site. However, the site has a wide variety of rock and soil types, with nonuniform distributions, as shown in Table 1.

## 3. Free-Field Vibration Test

To investigate the vibration transmissibility in soft soil foundations and verify the efficacy of the proposed approach, a comprehensive understanding of the existing vibration sources at construction sites was acquired to evaluate the vibration levels in silty clay layers at the project site, establishing a foundation for anti-microvibration design. Additionally, vibration levels in silty clay layers under varying loading conditions were measured to inform the anti-microvibration design process. Field tests were carried out in a representative soft soil region to support this investigation. Table 2 displays the primary instruments utilized in the microvibration tests.

**Figure 1:**
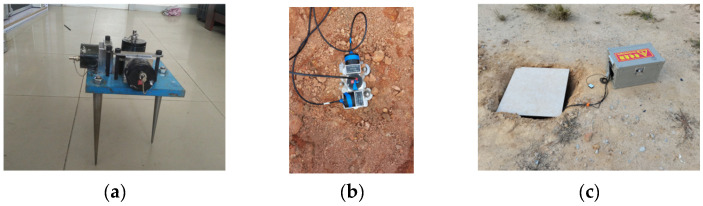
The installation and use of the sensors. (**a**) There are sensors and fixed supports, with the support designed to ensure the stability of the sensors on the soft soil foundation. (**b**,**c**) On-site photographs.

Regarding the installation and use of sensors, as shown in Figure 1, the advantages of using the adopted device can be summarized as follows: (1) it is capable of installing three sensors simultaneously for testing soil surface vibrations in three directions, with an adjustable horizontal orientation; (2) it has good integrity, and the rigidity of each component is high, ensuring the consistency of soil vibration measurements at the corresponding position and within the entire device.

Considering that the test site is close to a highway, the test vibration sources are ground vibrations from different types of highway traffic loads. Given that small vehicles produce smaller vibrations than large vehicles, the selected types of highway traffic for this test were common heavy-duty vehicles, as shown in Table 3. Since highway traffic varies according to demand, the load also varied. Furthermore, considering the nearby highway speed limit of 60 km/h, the vehicle speeds in the experiment were set to 30 km/h, 40 km/h, 50 km/h, and 60 km/h.

According to the site conditions, two test series were designed, as shown in Table 4. In the early-morning hours, fewer people are traveling, corresponding to series A; the remaining test period accounts for the environment corresponding to series B.

The sampling frequency was 256 Hz, and the sampled data represent the vibration velocities. A triaxial sensor was put in place at each measurement point in the vertical, axial (east–west), and transverse directions (north–south). The data collection was conducted using the software DASP-V11 Professional Edition—Vibration Noise Strain Impact from Beijing Institute of Vibration and Noise Technology. The data processing was performed using MATLAB.

In recent years, many researchers worldwide have proposed various vibration standards. For instance, in the context of environmental vibration monitoring, steady-state vibrations generally encompass environmental vibrations induced by rotating machinery and reciprocating machinery. The measurement parameter utilized is the equivalent continuous Z vibration level. Similarly, when implementing vibration isolation systems for precision instruments and equipment, restrictions are placed on the allowable linear displacement and linear velocity of vibrations based on specified regulations and standards tailored to the requirements of different instruments. Currently, the standard curve developed by Bolt Beranek and Newman (BBN) has been widely accepted and used as an evaluation criterion. Many enterprises adopt this criterion to control the installation conditions when defining anti-vibration indicators for their products. This paper also evaluates vibration standards based on this criterion.

## 4. Analysis of the Test Data

To reduce the impact of frequency leakage and boundary effects on the data, a computationally simple and convenient Hanning window was used for windowing. Furthermore, it is essential to note that the scientific device itself does not generate vibrations and is highly sensitive to environmental vibration frequencies, typically ranging from 1 to 100 Hz [35]. The vibration amplitude exhibits a substantial variation within the frequency range of 1–100 Hz, which is significantly greater than that of other frequency bands. Consequently, filtering is employed prior to numerical analysis to eliminate noise and interference frequency components, thereby enabling a more comprehensive analysis of the typical signal characteristics. To this end, in this paper, we adopted windowing and filtering to preprocess the collected data.

Regarding the three-directional signals acquired, the vertical vibration response is notably greater than the horizontal vibration response. Given that a traffic load primarily impacts the vertical vibration, this paper primarily focuses on the analysis of vertical data. This approach allows for a more targeted and precise investigation of the vibration effects considered and will ultimately provide a reference for site selection and design in subsequent laboratories.

### 4.1. Vibration Response of Soil under Pulsation

**Figure 2:**
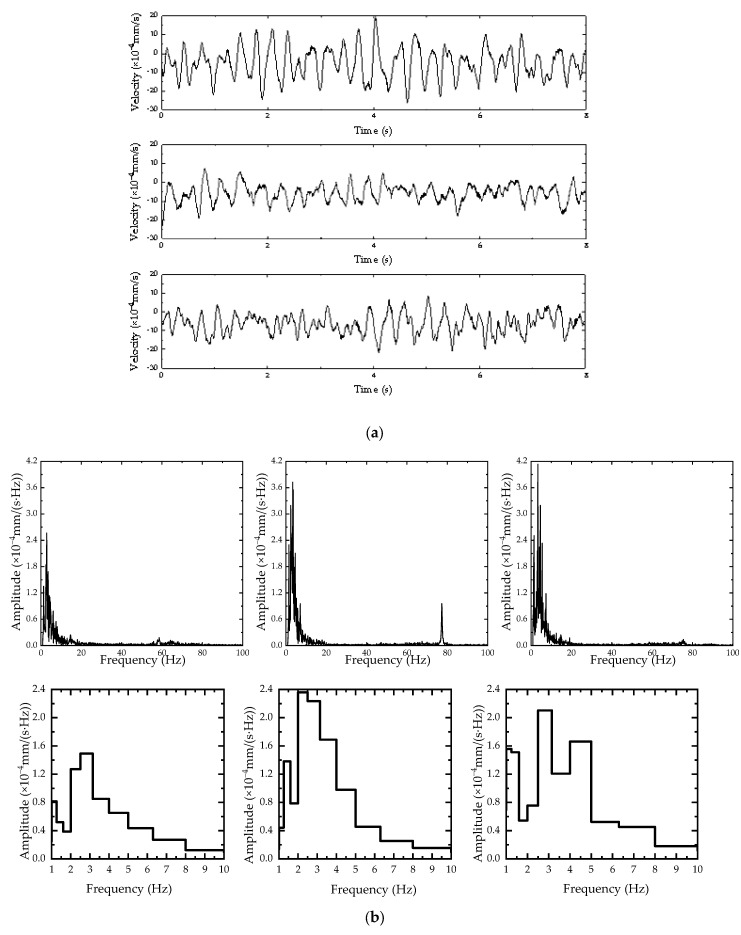
(**a**) Time-domain curves of ground pulsation; (**b**) frequency-domain curves of ground pulsation.

To ensure the accuracy of the test, the analysis was conducted during the early-morning hours when no vehicles were passing by. The overall sampling time was 30 min, with 8 s randomly selected from each minute for numerical analysis. Taking three sets of data as examples, the vibration velocity time-domain and frequency-domain curves of ground vibrations are shown in Figure 2. The laboratory vibration control standard predominantly involves the VC curve. Therefore, the subsequent analysis is primarily based on the VC curve.

According to the above figure, the amplitude of the ground vibration is 3 × 10^−4^ mm/s, and the dominant frequency is 2.2 Hz.

According to the seismic design code and references, layered soil in a foundation can be assumed to be a single soil mass to determine the natural frequencies of the layered elastic half-space foundation soil.

(1) $$f=C_{S}/(4H)$$

(2) $$C_{S}=\sum_{i=1}^{n}\left(C_{S_{i}}\times H_{i}\right)/H$$

where f is the natural frequency of the foundation soil. $C_{S}$ is the equivalent shear wave velocity, and $C_{S_{i}}$ is the shear wave velocity of the i-th layer of soil. H represents the total thickness of the foundation soil, but it should not exceed 20 m, while $H_{i}$ is the thickness of the i-th layer of soil.

To complete the initial survey, five survey pile locations were planned at the site where the laboratory will be constructed, with a geomagnetic pulse test near pile No. 2. According to the above formula, the natural frequency of the foundation soil at pile No. 2 can be calculated as 2.37 Hz.

Based on the above content, the vibration response characteristics of soft soil foundations can be understood. Due to the particularity of soft soil foundations, it is necessary to fully consider their differences when dealing with foundations. Therefore, the effectiveness of conventional foundation treatment methods for soft soil foundations needs to be explored. In the future, this can serve as a reference for focused discussion on the treatment methods for soft soil foundations.

### 4.2. Soil Vibration Response under the Influence of a Vehicle Driving on a Road

**Figure 3:**
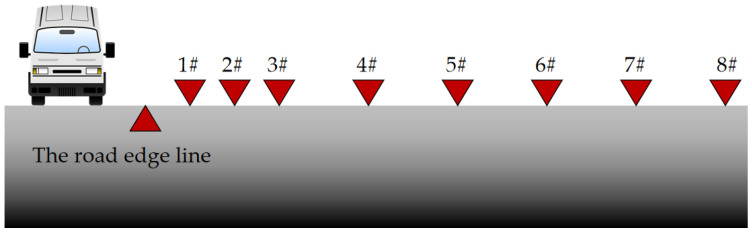
The arrangement of vibration sensors under condition B.

To study the vibration attenuation law of soft soil foundations under the influence of highway traffic loads, a vibration test line perpendicular to the road was arranged in the field. Eight test points were set up along the line at distances of 30, 45, 60, 90, 120, 150, 180, and 210 m from the road (see Figure 3). To avoid randomness in the test results, each control group was tested four or six times. Multiple sets of results were used to ensure the accuracy and effectiveness of the tests.

#### 4.2.1. Time Domain

**Figure 4:**
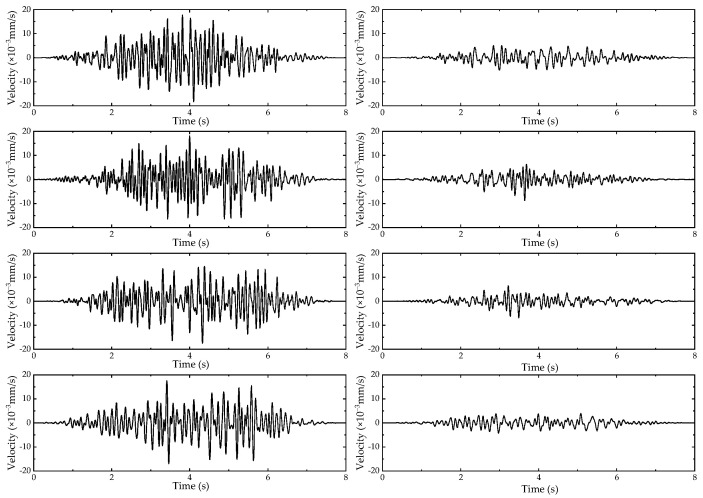
The time-domain results for vehicle type C (from top to bottom, the velocity decreases from 60 km/h to 30 km/h). The left figure corresponds to 60 m, and the right figure corresponds to 150 m.

**Figure 5:**
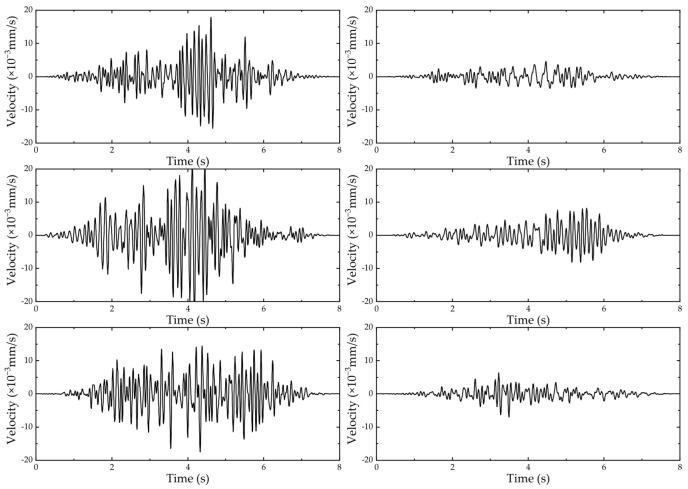
The time-domain results at 40 km/h (from top to bottom, the figure shows cases A, B, and C). The left figure corresponds to 60 m, and the right figure corresponds to 150 m.

The sample length before and after the vehicle passage was reduced to 8 s. To analyze the influence of different vehicle speeds on vibration, vehicle type C was taken as an example, and its velocity time-domain curve under different working conditions is shown in Figure 4. To consider the influence of different vehicle types on vibration, taking 40 km/h as an example, the corresponding velocity time-domain curves under different working conditions are shown in Figure 5.

At different vehicle operating speeds, the vibration amplitude at the same point shows a similar trend before and after the vehicle passes through. The specific amplitude peak values vary with the change in vehicle speed. This indicates that the different driving speeds of vehicles do not have a significant impact on the response of the foundation vibration.

For different vehicles with the same operating speed, the vibration amplitude at the same point shows a similar trend during the time before and after the vehicle passes through. The specific amplitude peak values vary with the change in vehicle type. This indicates that the difference in vehicle types does not have a significant impact on the response of foundation vibration.

To better analyze the valid information in the data, an exploration was conducted using a representative root mean square (RMS), enabling the conclusions to be more stable and facilitating the comparison of signal changes and fluctuations. To achieve this, the RMS of the vibration velocity was calculated by the following formula for each sample in the time domain.

(3) $$V_{RMS}=\sqrt{\frac{1}{N}\sum_{i=1}^{N}{\left[V(i)\right]}^{2}}$$

where $V_{RMS}$ is the RMS of the vibration velocity. N is the sample size. V(i) is the vibration velocity of the i-th sample.

**Figure 6:**
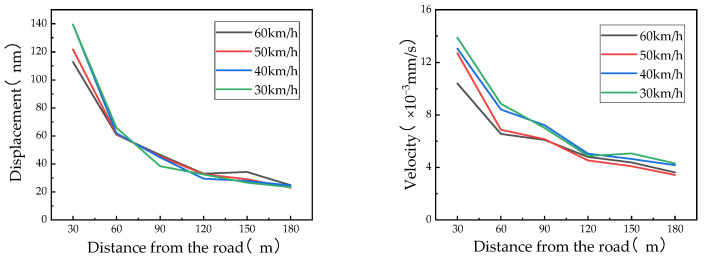
Vibration displacement and velocity in the vertical direction for the RMS analysis in the time domain with different speeds for vehicle type C.

**Figure 7:**
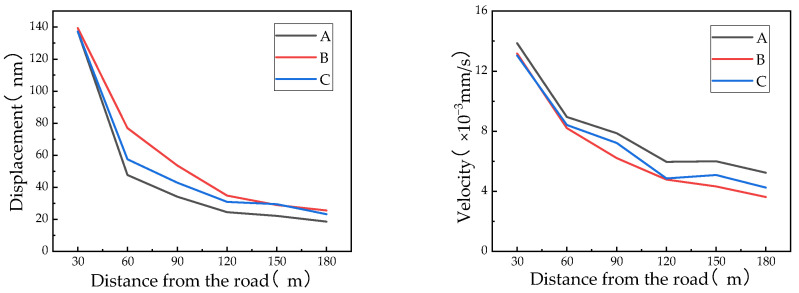
Vibration displacement and velocity in the vertical direction for the RMS analysis in the time domain for different vehicles at 40 km/h.

The attenuation characteristics under different vehicle types at the same speed and under the same vehicle type at different speeds are shown in Figure 6 and Figure 7, respectively.

According to the above figure, the main characteristics of vibration under different operating conditions are as follows:

(1) Under any working condition, the vibration time-domain results at the same test point exhibit little difference, indicating that the soil vibration response is less affected by the vehicle speed and type. The RMS of the time-domain analysis revealed that the influence of the vehicle speed and type on the vibration response is minimal, which is consistent with the above conclusion.
(2) As the distance from the vibration source increases, the vibration in the soil attenuates, with the attenuation amplitude in the near field being significantly greater than that in the far field. This pattern arises because the geometric damping and material damping of the soil dissipate the energy of the vibration during propagation until the vibration reaches a point where the energy is almost completely dissipated.
(3) Compared to that under the operating conditions of series A, the vibration velocity under the operating conditions of series B is significantly faster. This indicates that the site is sensitive to vehicle vibration; thus, vehicle vibration has a significant impact on the site and the instability of the soft soil foundation. To better design and plan the laboratory, it is necessary to comprehensively evaluate the safety of the site and carry out foundation treatments.

Consequently, the vibration response characteristics of soft soil subgrades under traffic loads can be understood. Therefore, future transportation planning and traffic control in this area should be arranged based on this.

#### 4.2.2. Frequency Domain

**Figure 8:**
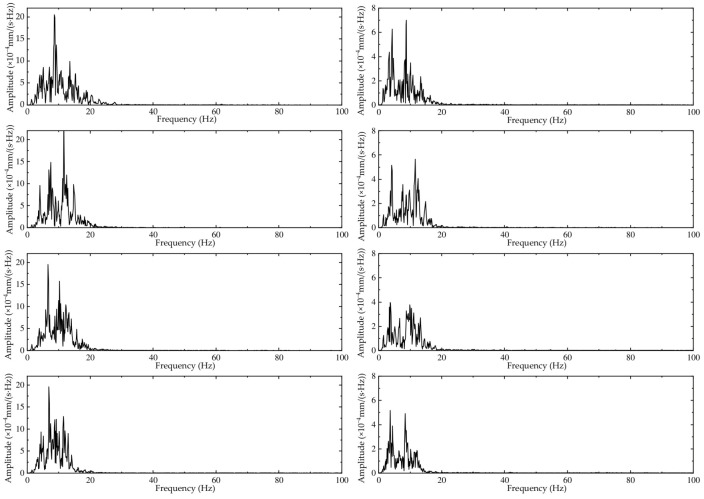
Frequency-domain results for vehicle type C (from top to bottom, the velocity decreases from 60 km/h to 30 km/h). The left figure is at 60 m, and the right figure is at 150 m.

**Figure 9:**
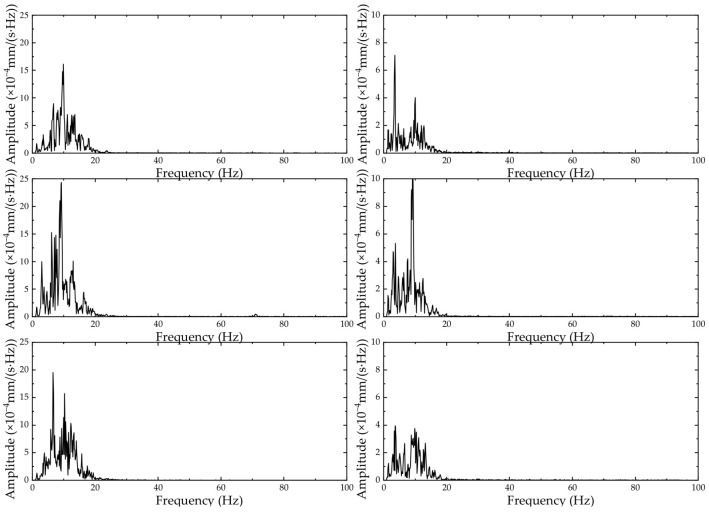
Frequency-domain results at 40 km/h (from top to bottom are A, B, and C). The left figure corresponds to 60 m, and the right figure corresponds to 150 m.

According to the time-domain analysis of Section 4.2.1, the corresponding frequency-domain curves are shown in Figure 8 and Figure 9.

The dominant frequency of vibration for vehicle type C at 60 m is concentrated at approximately 10 Hz, with a peak amplitude of 19~22 × 10^−4^ mm/s. At 150 m, the dominant frequency of vibration is concentrated between 3 and 10 Hz, with a peak amplitude of 3~7 × 10^−4^ mm/s. The vibrations at both points are concentrated in the low-frequency range and have higher amplitudes in the range of the Earth’s dominant frequency, indicating that passing vehicles are more likely to cause resonance with the Earth’s vibrations.

At the same speed, the dominant frequency of vibration for all three vehicle types at 60 m is concentrated at approximately 10 Hz, with a peak amplitude of 16~25 × 10^−4^ mm/s. At 150 m, the dominant frequency of vibration is concentrated between 3 and 10 Hz, with a peak amplitude of 3~12 × 10^−4^ mm/s. The vibrations at both points are concentrated in the low-frequency range and have higher amplitudes in the range of the Earth’s dominant frequency, indicating that passing vehicles are more likely to cause resonance with the Earth’s vibrations.

The figure shows that the main spectral peaks of the vibration velocity are within the range of 3–12 Hz, with two main peaks concentrated at approximately 3–5 Hz and 10–12 Hz. These frequencies are easily resonant with ground vibrations, and the spectral amplitude of the vibration velocity caused by traffic loads is significantly larger than that of ground vibrations. Therefore, the impact of vehicle loads on structural vibrations should be considered during site selection and design to avoid substantial increases in load effects.

#### 4.2.3. Vibration Transmissibility

Currently, the traditional definition of vibration transmissibility is the ratio between the response quantity of a linear system under zero initial conditions and the excitation quantity. However, for complex systems, it is difficult to measure the system response. Based on this, the ratio of the output quantities between systems is defined as the response transmissibility. In this study, the vibration transmissibility between two points is defined as the ratio of the amplitude of the vibration velocity calculated based on the one-third octave frequency band.

(4) $$Tr=\frac{X(f_{c})}{Y(f_{c})}$$

where Tr is the vibration transmissibility. $f_{c}$ is the center frequency of the one-third octave frequency band. X and Y are the vibration velocity amplitudes of two adjacent points.

**Figure 10:**
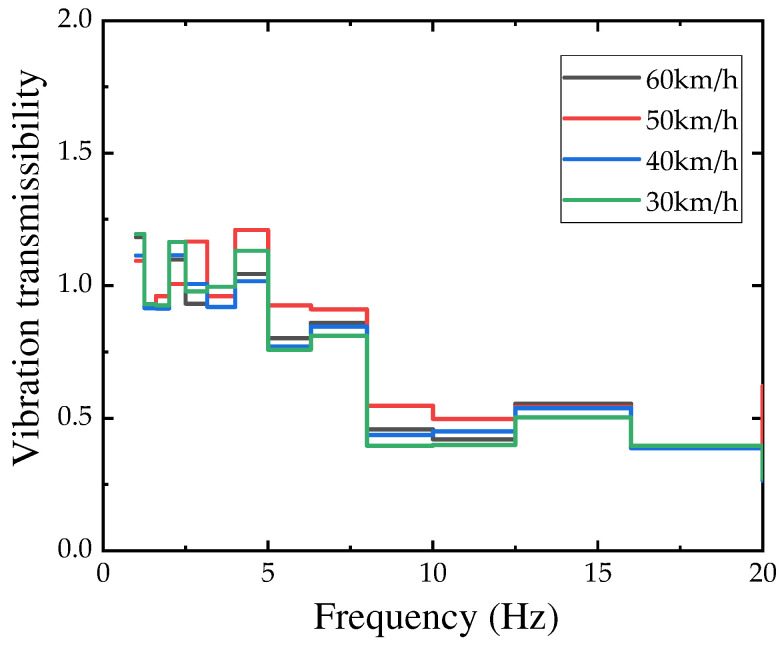
Vibration transmissibility for different speeds of vehicle type C.

**Figure 11:**
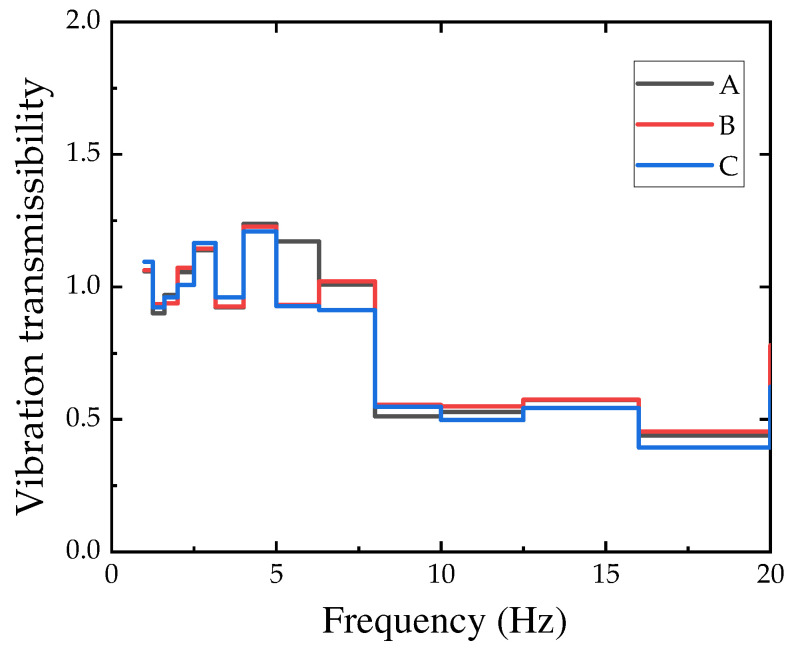
Vibration transmissibility for different vehicle types at 50 km/h.

Considering that the peak can reflect the inherent characteristics and boundary conditions of the vibration system, the amplitude and phase information of the vibration signal are relatively obvious, which is beneficial for understanding the dynamic characteristics of the vibration system. Therefore, the vibration transmission ratio within the frequency range of 1–20 Hz near the points is observed (Figure 10 and Figure 11).

From the figure, it can be seen that there is no obvious relationship between vibration transmission and vehicle speed and type. On this basis, the conventional vibration velocity at this site can be used for reference during the site’s construction design process.

### 4.3. Prediction of Soil Vibrations with a Vehicle Driving on a Road

**Figure 12:**
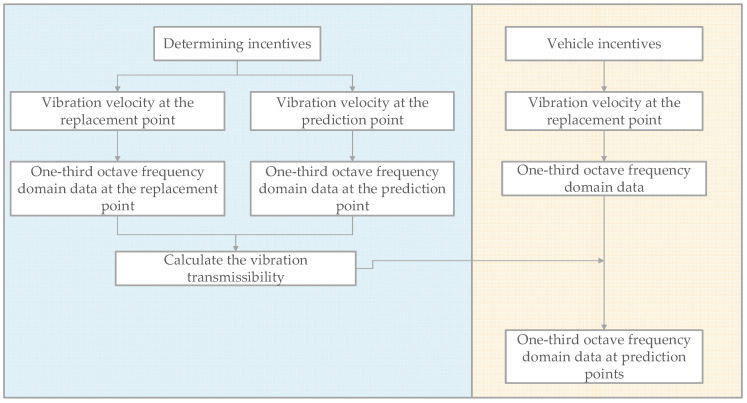
Forecasting framework chart.

Based on the calculated vibration transmission ratio and the known vibration response, the vibration response of other points can be predicted, as shown in Figure 12.

Under the determining incentives, the vibration velocities of the replacement point and the prediction point were measured, and the frequency-domain amplitudes of the two points at one-third octave were calculated to obtain the vibration transmissibility. By substituting this transmissibility into the one-third octave spectral data of the replacement point under vehicle incentives, the data of the prediction point were calculated.

**Figure 13:**
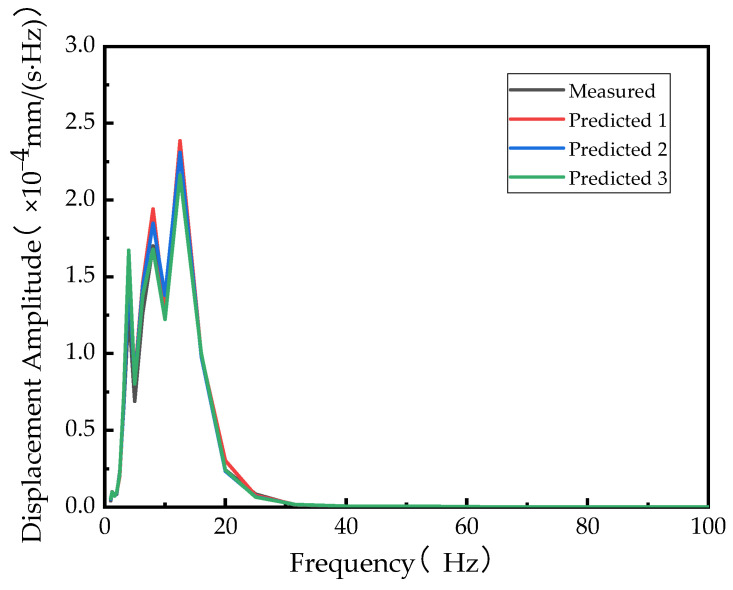
Projection curve for vehicle type C at 50 km/h obtained by using vibration transmissibility at different speeds.

**Figure 14:**
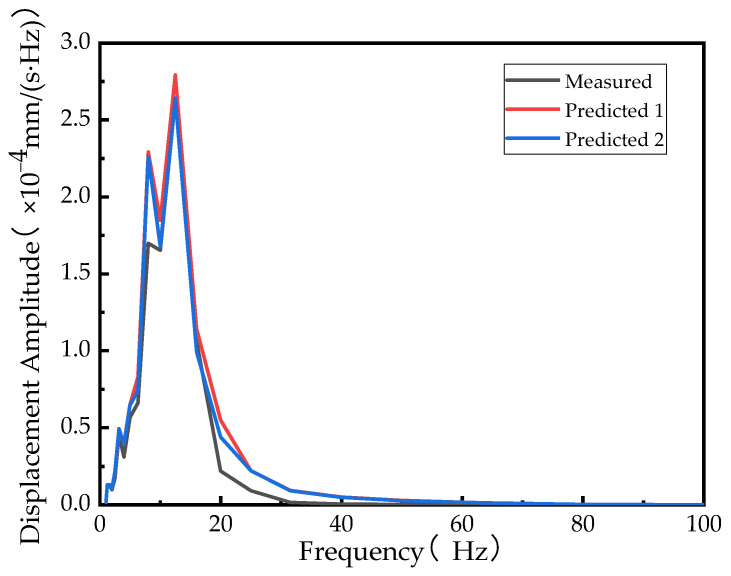
Projection curve for vehicle type A at 50 km/h obtained by using vibration transmissibility of different vehicles.

The vibration response of a vehicle at any speed can be obtained by the known vibration response at a certain speed. Similarly, by knowing the vibration response of any point on vehicle type A, the vibration response of other points on vehicle type A can be obtained by using the vibration transmissibility of vehicle type B or vehicle type C. Based on this, the prediction curves are shown in Figure 13 and Figure 14.

The predicted curves closely match the measured curves, indicating the presence of some potential interfering factors that cannot be completely ruled out. However, the calculated results are able to meet engineering requirements without the need for model establishment. Additionally, the calculation efficiency is high, making it applicable in a wider range of scenarios.

## 5. Conclusions

(1) The vibration response of soft soil foundations under traffic loads is primarily characterized by low frequencies. To prevent vibrations from affecting the progress of experiments, appropriate reinforcement measures should be taken to effectively reduce the transmission of vibration to the foundation. However, conventional foundation treatment methods may not necessarily be suitable for this specific type of foundation; hence, further research on vibration control for soft soil foundations is necessary in the future.
(2) Under traffic loads, the vibration response of soft soil foundations is prone to resonance at the natural frequency of the foundations. Therefore, this issue needs to be considered in the planning of construction sites and the control of traffic in nearby areas.
(3) The transmissibility between fixed points remains constant with changes in vehicle type and speed. The effectiveness and accuracy of the vibration prediction method based on transmissibility have been verified, providing guidance for the implementation and design of soft soil foundations, offering direction for microvibration control, and suggesting layouts for subsequent laboratory setups.

## Figures and Tables

**Table 1:** The conditions of the soil layers.

Number of Soil Layers	Average Thickness of Soil Layers (m)	Compressive Modulus ^1^ (MPa)	Bearing Capacity ^2^ (kPa)	Shear Wave Velocity ^3^ (m/s)
① Plain fill	0.5	4	70	120
② Mucky soil	3.2	4.7	60	95
③ Silty clay	15	11.9	180	230
④ Coarse sand	3.6	20	230	280
⑤ Residual gravel clay soil	1.5	12.2	200	260
⑥ Completely decomposed granite	0.9	22	300	300
⑦ Weathered granite	1.1	65.8	580	550
⑧ Moderately weathered granite	The burial depth of the top layer changes greatly	Excluding compression	3000	>800

^1^ Compressive modulus: The compressive modulus is a parameter that describes the ability of soil to resist deformation under pressure. Understanding the compressive modulus of soft soil can predict the settlement of the soil and ensure the stability of the engineering project. ^2^ Bearing capacity: The bearing capacity is the maximum load that soil can withstand. By evaluating the bearing capacity of soft soil, we can ensure the safety of the engineering project. ^3^ Shear wave velocity: The shear wave velocity is the speed at which shear waves propagate in soil and reflects the compactness and elastic modulus of soil.

**Table 2:** The main instruments used for testing.

Number	Instruments Name	Photograph	Instrument Model	Parameter
1	vibration transducer	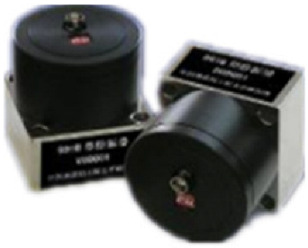	941B; Institute of Engineering Mechanics, CEA.	Sensitivity: 23v·s/m; Frequency response: 0.25–100 Hz.
2	data acquisition instrument	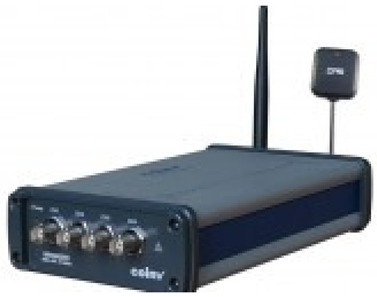	INV3062T; China Orient Institute of Noise & Vibration.	bit Delta-Sigma acquisition; 25–120 dB dynamic range; 4-channel parallel.

**Table 3:** Vehicle introduction.

Vehicle Type	Photograph	Load
A	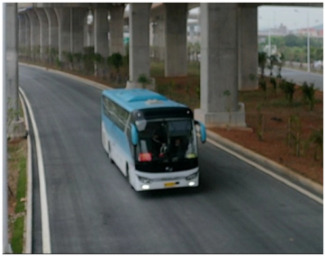	Lighter
B	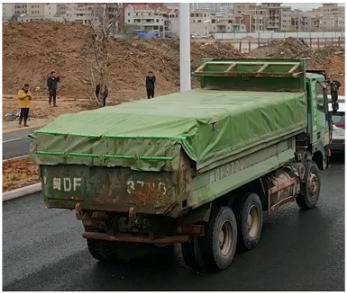	Heavier
C	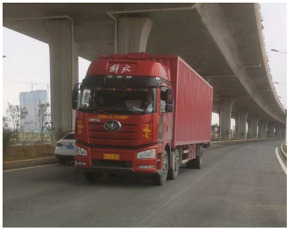	Moderately heavy

**Table 4:** Test conditions.

Content	Series	Test Condition	Description
Vibration testing	A	Ground pulsation	Excellent frequency and amplitude of soil vibration due to ground pulsation at different depths, with no interference from other vibration sources in the test area.
	B	Vibration response of soil under the influence of highway traffic loads	The vibration response and the attenuation characteristics under different traffic loads with no interference from other vibration sources in the site area.

## Data Availability

The data presented in this study are available upon request from the corresponding author.
